# Food safety knowledge, attitudes, and behavior of street food vendors and consumers in Handan, a third tier city in China

**DOI:** 10.1186/s12889-019-7475-9

**Published:** 2019-08-16

**Authors:** Lihua Ma, Hong Chen, Huizhe Yan, Lifeng Wu, Wenbin Zhang

**Affiliations:** 1 School of Management Engineering and Business, Hebei University of Engineering, Handan, 056038 People’s Republic of China; 2 School of Management, China University of Mining and Technology, Xuzhou, 221116 People’s Republic of China

**Keywords:** Street food, Food safety knowledge, Food safety attitude, Food safety behavior, Vendors, Consumers, Handan city

## Abstract

**Background:**

Food safety has long been the subject of scholarly research, and street food is a weak link in food safety supervision. Street food not only provides convenience for many people, but is also the livelihood for millions of low income people, making a great contribution to the economy of many developing countries.

**Methods:**

Street food safety is essential, and yet it has been rarely studied in China. Therefore, a typical city in China was selected as the research object to assess food safety knowledge, attitudes, and street food suppliers and consumer behaviors using questionnaires based on previous studies, and considering China’s particular characteristics and reasonable impacts identified in previous studies, such as increased income, work experience, licenses, and locations. The food safety knowledge and attitude questionnaire conformed with the national conditions in China. It was used to assess the food safety knowledge and attitudes toward food suppliers and consumers, where three main areas were addressed in the surveys and statistical analysis, as follows. (1) Statistical information including gender, age, education, income, food safety training, and specific elements related to the work experience of suppliers. (2) Knowledge of food safety including the awareness of consumers and suppliers regarding food poisoning pathogens, food and personal hygiene, high-risk groups, and correct cleaning. (3) A list of food handling behaviors was used to determine the behaviors and characteristics of subjects.

**Results:**

The results show that street food suppliers have generally poor food handling practices, and most are operating under unsanitary conditions. Food safety knowledge of street vendors in the High-tech Industries Development Zone was the lowest, most likely because these regions are located in rural-urban fringe zones, where education levels are generally relatively low. Food safety attitudes of the youngest consumers were significantly better than those of older age groups. Their educational level was also different, with correspondingly relatively high income for younger individuals. Most vendors chose locations near schools or supermarkets. Consumers and street food vendors had good understanding of food safety, but street vendors were relatively poor in carrying out safe food handling, with only 26.7% using or being fully equipped withhand-washing facilities, although more than 60% of vendors wore clean and tidy clothes and masks.

**Conclusions:**

Street food vendor training should be prioritized to improve the safety of street food. Other policies and measures should also be propagated to improve the food safety knowledge, attitudes, and behavior of vendors in Handan. Steps should be taken to improve street food stall operating conditions and facilities, including providing clean protected structures, access to potable water, and efficient waste collection and disposal systems. These findings should encourage government agencies to further promote strategies to improve street food safety.

**Electronic supplementary material:**

The online version of this article (10.1186/s12889-019-7475-9) contains supplementary material, which is available to authorized users.

## Background

Food safety has been the subject of research [1], and some have promoted it to the level of a national security issue [2]. Street food is a weak link in food safety supervision [3]. Street food is defined by the Food and Agriculture Organization (FAO) as “ready-to-eat foods and beverages sold and prepared by vendors or hawkers in streets or other public places” [4]. Street food provides a convenient diet for many people in developing countries [5, 6], and approximately 2.5 billion people eat street food every day, with the consumption supporting the livelihood of millions of low income people and contributing greatly to the economy [7].

Street food safety remains a major concern in developing countries, including China [8]. China’s food culture has a long history of street food. Most cities provide street food for locals and tourists, and street food has become part of the characteristic Chinese culture. Therefore, street food safety has become a matter of safety concern, and has been shown to be served in poor food handling and unsanitary conditions [9]. Most street food vendors are relatively uneducated and often uninformed, and have little effective regulatory or supervisory oversight [10]. In some developing countries [11], street food has been associated with outbreaks of foodborne diseases [12]. High levels of coliform bacteria have been found in street food in several countries [13], and street food has been identified as a common medium for transmission of antimicrobial-resistant pathogens [14].

There is an urgent need for research on street food safety and particularly in China, which appears to have rarely been the focus of previous research in this field. Therefore, a typical Chinese city was selected, and a suitable questionnaire was devised to assess food safety knowledge, attitudes, and street food vendor and consumer behaviors, considering China’s particular characteristics, and previously identified impacts, such as increased income, work experience, licenses, locations, etc. [9, 15]. The results showed that there is cause for significant concern for street food safety, and the development of improved and more effective strategies are needed.

## Methods

Handan is a typical third-tier city in China, and the regional central city for a key construction area, which therefore is suitable for research on safety food [16–18]. Food safety qualification rates in the city are not stable and food safety is a major public health problem in this area. Therefore, the current study investigated food safety knowledge and attitudes of vendors and consumers of street food in Handan city from June to August 2016. The study included four districts: Hanshan (HS), Congtai (CT), Fuxing (FX), and High-tech Industries Development Zone (HIDZ), and included 100 street vendors, 240 consumers, and 90 street vending stalls (See Additional files 1, 2, 3 for details). The number of street vendors and consumers were evenly distributed between the four districts. Structured written questionnaires were used to assess food safety knowledge and attitudes of consumers and vendors, and a checklist was used to evaluate street vendor food handling behaviors.

In China, it is unnecessary to receive written consent of participants for structured written questionnaires. The Ethics Statement included in the questionnaire instructions clearly stated that only respondents who agreed to the instructions participated in the survey. All of the participants read and approved the statement before they participated in the survey. In order to dispel any misgivings related to the ethics statement, we also clearly explained the purpose of the study and included an academic use only statement at the beginning of the questionnaire before it was issued. This statement read as follows:

***“Please think seriously about whether to participate in this survey! If you are in agreement, we will assume that you agree to our using the information provided.”***

We employed a questionnaire, online survey, and mailing methods. The consenting participants completed the survey and the identities of all the subjects were kept strictly confidential.

### Food safety knowledge and attitudes questionnaire

Tables 5, 6 and 7 show the questionnaire details, which were designed on the basis of previous studies to assess food safety knowledge and attitudes towards food suppliers and consumers [15]. This was the first time such a questionnaire had been translated into Chinese for use in China, and some items were partially modified. Prior to applying the questionnaire for the study, it was assessed by 100 people to ensure rationality of the design, and various changes and improvements were made before adoption of the final version. The data provided by the top 100 respondents were used to revise the questionnaire. The data analyzed in the manuscript did not include those from these 100 respondents. The data analysis presented in the manuscript was based on the final revised questionnaire, including the responses from 100 street vendors, 240 consumers, and 90 street vending stalls. The questionnaire was organized into three main sections as follows:

- Demographic information. This section gathered details regarding sex, age, educational level, income, and training in food safety, with a specific element relating to work experience for vendors.
- Food safety knowledge. This section assessed consumer and vendor awareness of food poisoning pathogens, food and personal hygiene, high risk groups, proper cleaning, etc. There were 18 questions with three possible answers: “yes”, “no” and “do not know”. Each “yes” answer was awarded one point, with the other two answers awarded 0 points. Hence, a maximum of 18 questions could be attained in this section. Each question had a maximum of 100 points, where a score of less than 50 was considered to indicate a low level of food safety knowledge, 50–75 denoted a satisfactory level, and better than 75 was considered good. *-*Food safety attitudes. This section assessed food safety attitudes, including food specification, food placement, and personal hygiene issues. There were 16 questions with three possible answers: “yes”, “no”, or “do not know”, with one point awarded for “yes” and 0 points for both other answers.

To ensure appropriate consumers and street vendors were included in the survey, researchers focused on schools, markets, parks, residential communities, and people-intensive streets in the four districts. All respondents participated voluntarily, were over 15 years of age, and were selected randomly. After interviewing 100 respondents, the questionnaire was revised and the new edition used to interview a further 340 respondents for final analyses, including 100 street vendors and 240 consumers.

### Food handling behavior checklist

In addition to questioning the respondents, researchers observed the various food stalls in operation, and completed a checklist detailing food handling behaviors and characteristics. The checklist details are shown in Table 8, and covered six sections:

- Food stall details,
- Environment around the stall,
- Personal hygiene,
- Food storage facilities at the stall,
- Utensil maintenance, and
- Licensing.

### Statistical analysis

The data obtained from the questionnaires and observation checklists were analyzed using SPSS version 21.0, and then exported to Microsoft Excel to calculate the various scores. We used the following analysis categories:

- Age groups were18–25, 26–35, 36–45, 46–55, 56–60 and > 60 years;
- Scores were aggregated into ranges of < 50, 50–75, and > 75 points;
- Consumer income cut-offs were grouped at 2000, 4000, 6000, and 8000 Yuan/month; and vendor work experience was 1, 3, 5, and 8 years.

Descriptive analyses used mean, standard deviation, maxima, and minima for each age category. Scores were assessed according to age, education, location, income or work experience, sex, and food safety training. The two-sample *t*-test was used to compare data sets in terms of sex and training status. Comparisons of more than two groups were conducted by fixed effects analysis of variance. Data and residual normality were first tested using quantile–quantile plots or the Kolmogorov–Smirnov test, and variance equality was checked using the modified Levene test. Non-normally distributed data sets and those with sample size less than 30 were analyzed using the non-parametric Wilcoxon rank sum test for two category cases, i.e., sex (male or female), food safety training status (trained or untrained) etc., and the Kruskal-Wallis rank sum test was used when there were more than two categories, i.e., age groups, income level, work experience, location (district), and educational level. Statistically significant differences were based on 95% confidence limits, i.e., α = 0.05 or *p* < 0.05.

## Results

### Demographic characteristics of consumers and street food vendors in Handan

Table 1 summarize demographic characteristics of the 340 respondents (240 consumers and 100 vendors). Consumer age ranged from 18 to 72 years (mean = 30.95 ± 11.3 years, with 90% between 18 and 45 years of age, and almost half (44.6%) were 18–25 years of age; whereas vendor age was somewhat more restricted: 20–55 years of age, mean = 34.4 ± 8.2 years. Consumer education level showed 78.8% had attained high school, university, or postgraduate level education, but almost half (48.3%) have no food safety training. In contrast, 68% of vendors had achieved lower (illiterate, or primary or middle school level), 25% high school, and 6% university or postgraduate education levels. The majority of vendors (70%) had not received any food safety training. Consumer income showed 70% earned less than 4000 Yuan/month. Vendor work experience was 0.5–18 years with the mean = 3.8 ± 2.5 years.

**Table 1:** Demographic data for street food consumers /vendorsin Handan City

consumers			
Characteristic	Count	Mean ± SD	Range
Sex			
Female	113 (47.1%)		
Male	127 (52.9%)		
Age (year)			
18–25	107 (44.6%)	30.95 ± 11.3	18–72
26–35	63 (26.3%)		
36–45	39 (16.3%)		
46–55	19 (7.9%)		
56–60	8 (3.3%)		
> 60	4 (1.7%)		
Education			
Illiterate	8 (3.3%)		
Primary school	16 (6.7%)		
Middle school	27 (11.3%)		
High school	58 (24.2%)		
University	122 (50.8%)		
Postgraduate	9 (3.8%)		
Food safety training			
Yes	124 (51.7%)		
No	116 (48.3%)		
Income (Yuan/month)			
< 2000	100 (41.7%)		
2001–4000	68 (28.3%)		
4001–6000	44 (18.3%)		
6001–8000	5 (2.1%)		
> 8000	10 (4.2%)		
Location (district)			
Hanshan	60 (25%)		
Congtai	60 (25%)		
Fuxing	60 (25%)		
HIDZ	60 (25%)		
Total	240		
vendors			
Sex			
Female	66 (66%)		
Male	34 (34%)		
Age (year)			
18–25	20 (20%)	34.4 ± 8.2	20–55
26–35	29 (29%)		
36–45	42 (42%)		
46–55	9 (9%)		
56–60	0 (0%)		
> 60	0 (0%)		
Education			
Illiterate	15 (15%)		
Primary school	14 (14%)		
Middle school	39 (39%)		
High school	25 (25%)		
University	6 (6%)		
Postgraduate	1 (1%)		
Food safety training			
Yes	30 (30%)		
No	70 (70%)		
Work Experience (years)			
0–1	12 (12%)	3.8 ± 2.5	
1–3	27 (27%)		
3–5	43 (43%)		
5–8	14 (14%)		
> 8	4 (42%)		
Location (district)			
Hanshan	25 (25%)		
Congtai	25 (25%)		
Fuxing	25 (25%)		
HIDZ	25 (25%)		
Total	100		

*HIDZ* High-tech Industries Development Zone

### Food safety knowledge of street food consumers and vendors

Table 2 shows food safety knowledge of street food consumers in Handan. Consumers from the four districts averaged 61 points for food safety knowledge, which was appropriate. However, many consumers (20%, 48) had poor food safety knowledge (score <  50). There was no significant difference in food safety knowledge with regards to sex (*p* = 0.322), educational level (*p* = 0.621), or monthly income (*p* = 0.540).

**Table 2:** Consumer food demographics and food safety knowledge

Characteristic	Score				
	< 50	50–75	> 75	Mean ± SD	Range
Sex					
Female	21 (8.8)	72 (30)	20 (8.3)	62 ± 15	28–100
Male	27 (11.3)	78 (32.5)	22 (9.2)	60 ± 17	11–100
Age (year)					
18–25	18 (7.5)	71 (29.6)	18 (7.5)	61 ± 16	11–100
26–35	8 (3.3)	38 (15.8)	17 (7.1)	65 ± 16	11–100
36–45	9 (3.8)	25 (10.4)	5 (2.1)	60 ± 16	39–100
46–55	9 (3.8)	8 (3.3)	2 (0.8)	54 ± 13	33–89
56–60	3 (1.3)	5 (2.1)	0 (0)	52 ± 10	28–72
> 60	1 (0.4)	3 (1.3)	0 (0)	53 ± 8	28–61
Education					
Illiterate	1 (0.4)	5 (2.1)	2 (0.8)	63 ± 13	28–83
Primary school	4 (1.7)	10 (4.2)	2 (0.8)	59 ± 13	28–89
Middle school	8 (3.3)	13 (5.4)	6 (2.5)	62 ± 13	39–100
High school	14 (5.8)	35 (14.6)	9 (3.8)	59 ± 14	11–89
University	18 (7.5)	82 (34.2)	22 (9.2)	62 ± 16	11–100
Postgraduate	3 (1.3)	5 (2.1)	1 (0.4)	54 ± 12	28–83
Food safety training					
Yes	14 (5.8)	77 (32.1)	33 (13.4)	66 ± 16	33–100
No	34 (14.2)	73 (30.4)	9 (3.8)	56 ± 16	11–94
Income (Yuan/month)					
< 2000	17 (7.1)	68 (28.3)	15 (6.3)	61 ± 16	11–100
2001–4000	13 (5.4)	44 (18.3)	11 (4.6)	61 ± 14	28–100
4001–6000	10 (4.2)	22 (9.2)	12 (5)	63 ± 14	39–100
6001–8000	5 (2.1)	9 (3.8)	3 (1.3)	63 ± 13	39–100
> 8000	3 (1.3)	7 (2.9)	1 (0.4)	54 ± 12	11–78
Location (district)					
Hanshan	14 (5.8)	40 (16.7)	6 (2.5)	60 ± 13	28–94
Congtai	9 (3.8)	32 (13.3)	19 (7.9)	65 ± 16	11–100
Fuxing	6 (2.5)	46 (19.2)	8 (3.3)	61 ± 15	28–100
HIDZ	19 (7.9)	32 (13.3)	9 (3.8)	58 ± 15	11–100
Total	48 (20)	150 (62.5)	42 (17.5)	61 ± 16	11–100

Generally, younger consumers had higher levels of food safety knowledge, whereas older subjects had less knowledge, where the consumers aged 26–35 years had the highest level of knowledge and those aged 56–60 years had the lowest. However, there were significant differences between those in the groups aged 18–25 and 46–55 years (*p* = 0.045), 26–35 and 46–55 years (*p* = 0.005), and 26–35 and 56–60 years (*p* = 0.028).

Mean consumer education = 66 points, and mean food training = 56points.Thus, consumers need to continue to improve their food safety knowledge.

Table 3 shows vendor food safety knowledge in Handan. Mean vendor food safety score = 58 points, which was lower than that of consumers (Table 3). In particular, more than half (54%, 54) had mean scores of 50–75, which was significantly lower than consumers (62.5%), and reflected insufficient street vendor training on food safety, and supervision was not in place. Vendor food safety knowledge was significantly different for age (*p* = 0.001) and sex (*p* = 0.01), and university educated vendors had higher food safety knowledge (69 ± 11). There were no significant differences between vendors who had received food safety training (60 ± 16) and those who had not (56 ± 16) (*p* = 0.287). Generally higher education level implied higher food safety knowledge. Thus, a poor level of education associated with food handling and storage practices may increase the risk of street food contamination [19, 20].

**Table 3:** Vendor food safety knowledge demographics

Characteristic	Score				
	< 50	50–75	> 75	Mean ± SD	Range
Sex					
Female	18 (18)	21 (21)	5 (5)	53 ± 16	28–94
Male	10 (10)	33 (33)	13 (13)	61 ± 16	22–89
Age (year)					
18–25	7 (7)	8 (8)	5 (5)	58 ± 16	33–94
26–35	7 (7)	16 (16)	6 (6)	61 ± 15	28–89
36–45	11 (11)	24 (24)	7 (7)	56 ± 15	22–83
56–60	3 (3)	6 (6)	0 (0)	52 ± 11	28–72
> 60	0 (0)	0 (0)	0 (0)	0	
Education					
Illiterate	5 (5)	10 (10)	0 (0)	51 ± 10	33–72
Primary school	5 (5)	8 (8)	1 (1)	55 ± 13	28–78
Middle school	10 (10)	22 (22)	7 (7)	58 ± 16	22–94
High school	7 (7)	12 (12)	6 (6)	59 ± 16	28–89
University	1 (1)	2 (2)	3 (3)	69 ± 11	39–83
Postgraduate	0 (0)	0 (0)	1 (1)	–	50
Food safety training					
Yes	5 (5)	18 (18)	7 (7)	60 ± 16	22–89
No	23 (23)	36 (36)	11 (11)	56 ± 16	28–94
Work experience (year)					
0–1	5 (5)	4 (4)	3 (3)	59 ± 13	33–83
1–3	5 (5)	16 (16)	6 (6)	61 ± 16	28–94
3–5	11 (11)	24 (24)	8 (8)	57 ± 16	22–89
5–8	5 (5)	8 (8)	1 (1)	53 ± 13	28–78
> 8	2 (2)	2 (2)	0 (0)	49 ± 10	33–72
Location (district)					
Hanshan	4 (4)	14 (14)	7 (7)	64 ± 15	39–94
Congtai	5 (5)	18 (18)	2 (2)	57 ± 16	22–89
Fuxing	10 (10)	8 (8)	7 (7)	56 ± 16	28–89
HIDZ	9 (9)	14 (14)	2 (2)	53 ± 14	33–83

Work experience and level of knowledge of food safety were not significantly different (*p* = 0.451), but food safety knowledge was significantly lower in HIDZ district (53 ± 14) than other regions (*p* = 0.025). This area was generally associated with people with lower incomes and lower educational levels (Table 1).

Table 4 show the responses of the consumers and vendors to the food safety knowledge questions, respectively, thereby providing greater insights into those with the highest and lowest levels of food safety knowledge. Some important issues regarding specific questions are highlighted below.

**Table 4:** Consumer / Vendorfood safety knowledge responses

Question	Response (consumer)			Response (vendor)		
	Yes	No	Do not know	Yes	No	Do not know
1. Abortion in pregnant women can be induced by food-borne disease.	146 (60.8)	6 (2.5)	88 (36.7)	65 (65)	31 (31)	4 (4)
2. Bloody diarrhea can be transmitted by food.	110 (45.8)	21 (8.8)	109 (45.4)	53 (53)	37 (37)	10 (10)
3. Swollen cans can contain microorganisms.	179 (74.6)	13 (5.4)	48 (20)	78 (78)	5 (5)	17 (17)
4. During infectious disease of the skin, it is necessary to take leave from work.	198 (82.5)	9 (3.8)	33 (13.8)	74 (74)	6 (6)	20 (20)
5. Eating and drinking in the work place increase the risk of food contamination.	189 (78.8)	16 (6.7)	35 (14.6)	57 (57)	10 (10)	33 (33)
6. Hepatitis A virus is a foodborne pathogens.	103 (42.9)	15 (6.3)	122 (50.8)	43 (43)	7 (7)	50 (50)
7. Microbes are in the skin, nose and mouth of healthy handlers.	186 (77.5)	7 (2.9)	47 (19.6)	62 (62)	10 (10)	28 (28)
8. Salmonella is among the food-borne pathogens.	119 (49.6)	7 (2.9)	114 (47.5)	38 (38)	8 (8)	54 (54)
9. Staphylococcus is among the food-borne pathogens.	111 (46.3)	8 (3.3)	121 (50.4)	35 (35)	9 (9)	56 (56)
10. Influenza can be transmitted by aerosols rather than food.	169 (70.4)	18 (7.5)	53 (22.1)	56 (56)	20 (20)	24 (24)
11. Using gloves while handling food reduces the risk of food contamination.	181 (75.4)	17 (7.1)	42 (17.5)	81 (81)	10 (10)	9 (9)
12. Washing hands before work reduces the risk of food contamination.	212 (88.3)	5 (2.1)	23 (9.6)	77 (77)	10 (10)	13 (13)
13. AIDS can be transmitted by food.	113 (47.1)	73 (30.4)	54 (22.5)	32 (32)	39 (39)	29 (29)
14. Children, healthy adults, pregnant women and older individuals are at equal risk for food poisoning.	77 (32.1)	109 (45.4)	54 (22.5)	31 (31)	39 (39)	30 (30)
15. Food prepared in advance reduces the risk of food contamination.	162 (67.5)	17 (7.1)	61 (25.4)	68 (68)	10 (10)	22 (22)
16. Proper cleaning and sanitization of utensils decrease the risk of food contamination.	210 (87.5)	8 (3.3)	22 (9.2)	79 (79)	11 (11)	10 (10)
17. Reheating cooked foods can contribute to food contamination.	100 (41.7)	50 (20.8)	90 (37.5)	48 (48)	14 (14)	38 (38)
18. Washing utensils with detergent leaves them free of contamination.	115 (47.9)	58 (24.2)	67 (27.9)	40 (40)	31 (31)	29 (29)

Question (Q)1: Although more than 60% of consumers (60.8%) and vendors (65%) knew abortion in pregnant women could be induced by food-borne diseases, many consumers either believed otherwise or did not know (2.5 and 36.7%, respectively), whereas 31 and 4% of vendors either believed otherwise or did not know, respectively.

Q2: Only 45.8% of consumers knew that bloody diarrhea can be transmitted by food.

Q3: There was generally good understanding that swollen cans can contain microorganisms for both consumers (74.6%) and vendors (78%).

Q4: There was generally good understanding that it was necessary to take leave from work during infectious disease of the skin for both consumers (82.5%) and vendors (74%).

Q5: Most of the consumers (78.8%) were aware that eating and drinking in the work place increases the risk of food contamination, but the vendors (57%) were significantly less aware of this problem.

Q6, Q8, Q9: Less than half the consumers knew that hepatitis A (42.9%), Salmonella (49.6%), and Staphylococcus (46.3%) were pathogens responsible for food-borne diseases, but this was even less well known amongst vendors (43, 38, and 35%, respectively).

Q7: The vast majority of consumers (77.5%) and vendors (62%) knew that microbes were in the skin, nose, and mouth of healthy food handlers.

Q10: Many consumers (70.4%) and vendors (56%) wrongly believe that Influenza can be transmitted by food.

Q11, Q12: Almost all consumers (75 and 88.3%, respectively) and vendors (81 and 77%, respectively) knew that washing their hands and using gloves lowered the risk of food contamination.

Q12: Consumers (88.3%) and vendors (77%) have good understanding of reducing contamination risk by hand washing before work.

Q13: Many consumers (47.1%) and vendors (32%) wrongly believe that AIDS can be transmitted by food.

Q16: Almost all consumers (87.5%) and street vendors (79%) knew that proper cleaning and disinfection of food utensils reduced the risk of contamination.

Q17: Less than half of consumers (41.7%) and vendors (48%) believe that reheating cooked foods contributed to food contamination.

### Consumer and street vendor food safety attitudes

Table 5 shows consumer attitudes toward food safety. Consumers also had an adequate understanding of food safety (mean = 74.2%), where 83.3% had scores > 50 points and 20.4% had scores > 75 points.

**Table 5:** Consumer food safety attitude demographics

Characteristic	Score				
	< 50	50–75	> 75	Mean ± SD	Range
Gender					
Female	17 (7.1)	71 (29.6)	25 (10.4)	62 ± 15	6–100
Male	23 (9.6)	80 (33.3)	24 (10)	61 ± 15	19–100
Age (year)					
18–25	18 (7.5)	66 (27.5)	23 (9.6)	61 ± 15	6–100
26–35	9 (3.8)	41 (17.1)	13 (5.4)	63 ± 15	31–100
36–45	7 (2.9)	24 (10)	8 (3.3)	61 ± 15	38–81
46–55	3 (1.3)	13 (5.4)	3 (1.3)	58 ± 15	25–94
56–60	2 (0.8)	4 (1.7)	2 (0.8)	63 ± 15	19–100
> 60	1 (0.4)	3 (1.3)	0 (0)	55 ± 13	38–69
Education					
Illiterate	2 (0.8)	2 (0.8)	4 (1.7)	63 ± 15	38–81
Primary school	4 (1.7)	10 (4.2)	2 (0.8)	57 ± 15	18–94
Middle school	6 (2.5)	13 (5.4)	8 (3.3)	63 ± 15	25–100
High school	8 (3.3)	39 (16.3)	11 (4.6)	63 ± 15	31–94
University	17 (7.1)	84 (35)	21 (8.8)	61 ± 15	6–100
Postgraduate	3 (1.3)	3 (1.3)	3 (1.3)	60 ± 15	44–75
Food safety training					
Yes	17 (7.1)	78 (32.5)	29 (12.1)	64 ± 15	6–100
No	23 (9.6)	73 (30.4)	20 (8.3)	59 ± 15	19–100
Income (Yuan/monthly)					
< 2000	20 (8.3)	63 (%26.3)	17 (7.1)	60 ± 15	19–100
2001–4000	12 (5)	43 (17.9)	13 (5.4)	61 ± 15	31–88
4001–6000	2 (0.8)	28 (11.7)	14 (5.8)	68 ± 15	31–100
6001–8000	4 (1.7)	11 (4.6)	2 (0.8)	57 ± 15	6–75
> 8000	2 (0.8)	6 (2.5)	3 (1.3)	61 ± 14	39–81
Location (district)					
Hanshan	7 (2.9)	38 (15.8)	15 (6.3)	63 ± 13	31–88
Congtai	7 (2.9)	41 (17.1)	12 (5)	63 ± 14	25–100
Fuxing	11 (4.6)	40 (16.7)	9 (3.8)	60 ± 16	19–100
HIDZ	15 (6.3)	32 (13.3)	13 (5.4)	60 ± 18	6–100
Total	40 (16.7)	151 (62.9)	49 (20.4)	61 ± 15	6–100

Table 5 shows that increased education level significantly increased the proportion of consumers with score > 50 points.

Regarding income, we can see that an income of 4001 to 6000 Yuan for the consumer showed the highest was 68 ± 15 on food safety and safety attitude.

Tables 7 show customer and vendor attitudes towards food safety, respectively. Some important issues regarding specific questions are highlighted below.

Q3: More than 3/4 of customers and vendors (87.5 and 83%, respectively) believe that the temperature of the refrigerator/freezer should be regularly checked to reduce the risk of food contamination.

Q4: Consumers (85.5%) and vendors (78%) agree that worker health should be assessed before commencing work.

Q6, Q7, Q8: Only half of those questioned (51.3%) were able to identify wearing masks as an important practice for reducing contamination. However, wearing masks or wearing gloves or a hat were considered important measures to reduce the risk of food contamination, by consumers and suppliers and therefore regarded as important in food safety (64.2 and 63%, respectively).

Q15: Only 1/3 of consumers and vendors thought thawed food should not be refrozen and that raw meat should be stored on the bottom shelf in the refrigerator.

### Vendor food safety behavior

Almost half of the food stalls were open (46.7%), with 23.3 and 13.3% being covered or half covered, respectively. The environmental indicators around the booth were also observed.

Vendors generally did not wear jewelry while handling food (66.7%), smoke while handling food (70%), or re-use utensils without cleaning them to prepare food (57.8%). However, barely half stored raw and cooked or partially cooked food in sealed and separated containers, and they were generally poor at cleaning their utensils with only 1/3 using soapy water. More than 60% of the observed vendors had no operating or health permits or health certificates.

## Discussion

The findings show that consumers have appropriate levels of food safety knowledge and attitudes, whereas vendor knowledge is poor, which is also reflected in their largely inadequate facilities and unhygienic behavior while selling foods. Most consumers (78.8%) were educated to at least high school, whereas most of vendors had significantly lower education levels and had no formal food safety training. This would greatly contribute to vendor’s poor food safety knowledge levels, attitudes and unhygienic behavior.

Of particular concern, more than half the vendors (53.3%) did not wash their hands before handling, preparing, or serving foods, and 72.2% used bare hands during cooking. More than half (53.3%) the observed stalls did not have direct access to potable water and 73.3% operated without adequate hand washing facilities.

The number of male and female respondents in the street food consumer survey is 127 (52.9%) and 113 (47.1%), respectively. Compared with 72 (60%) and 48 (40%) in Literature 9 and 60 (37.5%) and 100 (62.5%) in Literature 15, this survey covers a larger number of respondents with relatively balanced ratio of males and females, which can better ensure its objectivity and rationality. In the meantime, for the item “Food safety training” in Tables 1, 51.7% of the respondents answer that they have received food safety training before, which is in sharp contrast with the 24.2 and 11.3% respectively in Literature 9 and 15, indicating that consumers have been paying more and more attention to food safety with the rapid development of China’s economy and increasing life quality standards of people in recent years.

As can be seen from Table 1, although the gender of consumers were relatively uniform (female 47.1%, male 52.9%), vendors were predominantly female (female 66%, male 34%), which is similar to previous developing country surveys, e.g. Literature 9 and 15. In literature 9 and 15, female vendors accounted for 72.5% (29/40) and 88.7% (71/80), indicating that males are less competitive in food processing than females. This is maybe because the female vendors are more reliable, safer, cleaner, friendlier and more patient for food consumers. However, it also can be seen in Table 1 that 30% of the respondents’ answer goes to “yes” to the item “Food safety training”. It can be found that food vendors are less recognized by consumers in this respect, but the ratio is still larger than the 5 and 21.3% respectively in Literature 9 and 15, which shall be focused by the relevant authorities.

Traditionally, females are more commonly responsible for housework in China. However, with societal changes, more females now work outside the home. The higher proportion of female vendors (66%) may be advantageous, since female street food vendors tend to provide higher quality nutrition than male counterparts [21]. A US study showed that female vendors tended to prepare safer food [22], and the vendors interviewed in that study were educated to at least high school level.

In Tables 4, 42.9% of the respondents’ answer goes to “Yes” to Q6, and 50.8% answer “Do not know”, indicating that people are lack of understanding of Hepatitis A virus and its harm extent. In fact, the heating sterilization for the food and the utensils to place food is an important way to effectively restrict the spread of Hepatitis A virus. In Literature 15, 84.4% of the respondents’ answer goes to “Do not know”, while 60% give the same answer in Literature 9. It can be concluded that consumers need to improve the relevant knowledge.

In Table 4, it is also found in the food vendors survey that 54% of them have little understanding of Hepatitis A virus, while the ratio is up to 72.5 and 98.7% respectively in Literature 9 and 15. As food safety guarantors, the attention paid by food vendors to food safety is far from enough, which requires the relevant government departments to strengthen the training for and knowledge dissemination to the food vendors. These results highlight that street food vendor training should be prioritized to improve street food safety.

Table 5 shows consumer attitudes toward food safety. The mean score = 61 ± 15, indicating that consumers have a good understanding of food safety, which is consistent with previous studies, such as one in Haiti [15].

The food safety attitudes of younger consumers (26–35 years, mean = 68 ± 15 years) were significantly higher than those of the members of the other age groups, which was consistent with the results obtained in previous studies [23]. In general, the younger consumers were better educated than the older consumers and were more receptive to new media, such as mobile phones, computers, and network communication training.

Regarding income, we can see that an income of 4001 to 6000 Yuan for the consumer showed the highest was 68 ± 15 on food safety and safety attitude, which is consistent with the education level and with previous studies. The different districts all had mean scores over 60 points, which supports the coordinated development of Handan city in the process of urban development.

Table 6 shows vendor food processing attitudes. Vendor food safety attitudes were superior to that of consumers. Vendor mean = 62 ± 16, indicating that vendors had a generally good understanding, with only 10% scoring < 50. China has been working on improving food safety, particularly with respect to stricter management, control, and supervision of vendors and producers [24].

**Table 6:** Vendor food safety attitude demographics

Characteristic	Score				
	< 50	Score:50–75	< 50	Mean ± SD	Range
Sex					
Female	5 (5)	31 (31)	8 (8)	63 ± 16	13–100
Male	5 (5)	37 (37)	14 (14)	62 ± 16	0–94
Age (year)					
18–25	3 (3)	11 (11)	6 (6)	60 ± 16	0–88
26–35	3 (3)	19 (19)	7 (7)	64 ± 15	13–100
36–45	4 (4)	31 (24)	7 (7)	63 ± 14	31–94
46–55	0 (0)	7 (7)	2 (2)	59 ± 9	50–81
56–60	0 (0)	0 (0)	0 (0)	0	
> 60	0 (0)	0 (0)	0 (0)	0	
Education					
Illiterate	1 (1)	11 (10)	3 (3)	61 ± 10	44–81
Primary school	1 (1)	10 (10)	3 (3)	61 ± 12	31–81
Middle school	2 (2)	29 (29)	8 (8)	64 ± 13	38–94
High school	4 (4)	14 (14)	7 (7)	64 ± 15	13–100
University	2 (2)	2 (2)	2 (2)	51 ± 16	0–88
Postgraduate	0 (0)	1 (1)	0 (0)	–	50
Food safety training					
Yes	4 (4)	19 (19)	7 (7)	59 ± 16	0–94
No	6 (6)	49 (49)	15 (15)	63 ± 15	13–100
Work experience (year)					
0–1	2 (2)	7 (7)	3 (3)	55 ± 15	0–88
1–3	4 (4)	15 (15)	8 (8)	65 ± 15	13–100
3–5	2 (2)	34 (34)	7 (7)	62 ± 13	31–94
5–8	2 (2)	8 (8)	4 (4)	65 ± 13	38–94
> 8	0 (0)	2 (2)	2 (2)	55 ± 5	50–63
Location (district)					
Hanshan	3 (3)	14 (14)	8 (8)	63 ± 12	38–88
Congtai	2 (2)	16 (16)	7 (7)	67 ± 12	44–94
Fuxing	5 (5)	16 (16)	4 (4)	57 ± 16	0–100
HIDZ	0 (0)	22 (22)	3 (3)	62 ± 11	50–94
Total	10 (10)	68 (68)	22 (22)	62 ± 16	0–100

In Table 7, 28.3% of the respondents’ answer goes to “Yes” for Q16, 38.3% answer “No”, and 33.4% answer “Do not know”, revealing that consumers are not sure whether the eggs they bought shall be washed immediately. In fact, washing eggs is not correct. Eggs, especially the shells of fresh eggs, have a layer of powdery gelatinous substances. The main function of these substances is to prevent bacteria from invading the inside of the eggs. Meanwhile, it also prevents the water in the eggs from evaporating and protects the egg whites and egg yolks. If we wash away these gelatinous substances, the eggs will become easily deteriorated and difficult to preserve. Therefore, do not use water to clean the eggs after they are bought. If you think that there are too many dirty things in the egg shell, you can wipe them off with a dry rag.

**Table 7:** Consumer / Vendorfood safety attitude responses

Question	Response (consumer)			Response (vendor)		
	Yes	No	Do not know	Yes	No	Do not know
1. Proper hand hygiene can prevent food-borne diseases.	198 (82.5)	13 (5.4)	29 (12.1)	81 (81)	5 (5)	14 (14)
2. Raw and cooked foods should be stored separately to reduce the risk of food contamination.	197 (82.1)	6 (2.5)	37 (15.4)	83 (83)	2 (2)	15 (15)
3. It is necessary to check the temperature of refrigerators/freezers periodically to reduce the risk of food contamination	210 (87.5)	2 (0.8)	28 (11.7)	83 (83)	4 (4)	13 (13)
4. The health status of workers should be evaluated before employment	206 (85.8)	9 (3.8)	25 (10.4)	78 (78)	6 (6)	16 (16)
5. The best way to thaw a chicken is in a bowl of cold water	112 (46.7)	31 (12.9)	97 (40.4)	51 (51)	9 (9)	40 (40)
6. Wearing masks is an important behavior to reduce the risk of food contamination	190 (79.2)	15 (6.3)	35 (14.6)	83 (83)	5 (5)	12 (12)
7. Wearing gloves is an important behavior to reduce the risk of food contamination	184 (76.7)	17 (7.1)	39 (16.3)	78 (78)	8 (8)	14 (14)
8. Wearing caps is an important behavior to reduce the risk of food contamination	154 (64.2)	38 (15.8)	48 (20)	63 (63)	14 (14)	23 (23)
9. Dish towels can be a source of food contamination	117 (48.8)	51 (21.3)	72 (30)	57 (57)	9 (9)	34 (34)
10. Knives and cutting boards should be properly sanitized to prevent cross contamination	196 (81.7)	12 (5)	32 (13.3)	78 (78)	9 (9)	13 (13)
11. Food handlers who have abrasions or cuts on their hands should not touch foods without gloves	188 (78.3)	17 (7.1)	35 (14.6	72 (72)	17 (17)	11 (11)
12. Well-cooked foods are free of contamination	60 (25)	121 (50.4)	59 (24.6)	31 (31)	44 (44)	25 (25)
13. Can a closed can/jar of cleaning product be stored together with closed cans and jars of food products	122 (50.8)	43 (17.9)	75 (31.3)	52 (52)	12 (12)	36 (36)
14. Defrosted foods can be refrozen	82 (34.2)	78 (32.5)	80 (33.3)	30 (30)	37 (37)	33 (33)
15. The ideal place to store raw meat in the refrigerator is on the bottom shelf	75 (31.3)	50 (20.8)	115 (47.9)	34 (34)	23 (23)	43 (43)
16. Eggs must be washed after purchase as soon as possible	68 (28.3)	92 (38.3)	80 (33.3)	27 (27)	44 (44)	29 (29)

Table 8 shows observed street food facility characteristics and vendor food handling behaviors. Almost 1/3 of the vendors were near schools, and 22.2% near malls, which seems sensible, since these locations will have the largest personnel flow (e.g. Hebei University of Engineering has almost 30,000 students and teachers), consistent with a previous study [17].

**Table 8:** Observed food handling behavior and characteristics of vending sites in Handan

Observed item	Practice	
	Yes	No
Facilities		
1. Stall location		
Near school	28 (31.1)	0 (0)
Near supermarket	20 (22.2)	0 (0)
Farmer’s market	15 (16.7)	0 (0)
Street corner	8 (8.9)	0 (0)
Near residence community	13 (14.4)	0 (0)
Mobile vendor	5 (5.6)	0 (0)
Other	1 (1.1)	0 (0)
2. What material is the structure made of where the food was sold:		
Stainless steel	44 (48.9)	0 (0)
Zinc/iron	22 (24.4)	0 (0)
Plastic	9 (10)	0 (0)
Wooden table	8 (8.9)	0 (0)
Other	7 (7.8)	0 (0)
3. What structure is the facility where the food was prepared:		
Covered	21 (23.3)	0 (0)
Canopy	42 (46.7)	0 (0)
Semi-closed container	12 (13.3)	0 (0)
Container	6 (6.7)	0 (0)
Uncovered	9 (10)	0 (0)
4. Where was the food prepared:		
On site	71 (78.9)	0 (0)
At home	19 (21.1)	0 (0)
Environment around the stall		
5. Environment around the stall is clean	61 (67.8)	29 (32.2)
6. Access to potable water at the site or close to the site	42 (46.7)	48 (53.3)
7. Adequate hand washing facilities available	24 (26.7)	66 (73.3)
8. Adequate waste water or food disposal facilities available	41 (45.6)	49 (54.4)
9. Stall is far from rubbish	75 (83.3)	15 (16.7)
10. Stall is far from waste water	69 (76.7)	21 (23.3)
11. Stall is far from toilet facilities and open drains	65 (72.2)	25 (27.8)
12. Stall is far from animals	67 (74.4)	23 (25.6)
13. No flies on the stall	47 (52.2)	43 (47.8)
Personal hygiene		
14. Operator washed their hands in clean water each time before handling, preparing, or serving of food	48 (53.3)	42 (46.7)
15. Operator washed their hands each time after visiting the toilet	54 (60)	36 (40)
16. Operators clothes were clean and presentable	77 (85.6)	13 (14.4)
17. Operator used an apron when handling, preparing, or serving food	64 (71.1)	26 (28.9)
18. Operator handled food with bare hands	65 (72.2)	25 (27.8)
19. Operator nails were clean and short	71 (78.9)	19 (21.1)
20. Operator hair was covered when handling, preparing, or serving food	39 (43.3)	51 (56.7)
21. Operator wore a mask when handling, preparing, or serving food	36 (40)	54 (60)
22. Operator handled money while serving food	52 (57.8)	38 (42.2)
If answer to Q22 is yes: did operator wash their hands after handling money before handling food again?	12 (23.1)	40 (76.9)
23 Dirt or dust was removed using		
an apron	20 (22.2)	0 (0)
bare (uncovered) hands	15 (16.7)	0 (0)
dirty cloth	27 (30)	0 (0)
clean cloth	28 (31.1)	0 (0)
24. Operator wore jewelry while handling food	30 (33.3)	60 (66.7)
If answer to Q24 is yes: was the jewelry adequately covered	6 (20)	24 (80)
25. Operator smoked while handling food	27 (30)	63 (70)
26. Operator used the same utensils to prepare raw and cooked food	49 (54.4)	41 (45.6)
Food storage		
27. Food was stored/displayed in sealed containers	50 (55.6)	40 (44.4)
28. Raw, partially cooked, and cooked food products were kept separate	59 (65.6)	31 (34.4)
29. Previously cooked foods were kept cool e.g. in an ice box or refrigerator	41 (45.6)	49 (54.4)
Utensils		
30. Utensils were covered	46 (51.1)	44 (48.9)
31. Utensils were adequately cleaned every time after use	52 (57.8)	38 (42.2)
32. Utensils were cleaned with soapy water	33 (36.7)	57 (63.3)
License		
33. Operator had a license	35 (38.9)	55 (61.1)
34. Operator had a hygiene license	35 (38.9)	55 (61.1)
35. Operator had a health certificate	41 (45.6)	49 (54.4)

Almost half of the street vendors (48.9%) chose stainless steel cooking materials, believing that stainless steel was safer and healthier than other options. This practice has improved and promoted food safety and health in China. However, 24.4% of vendors used iron dishes, which are unsafe because iron easily rusts, and iron rust is poisonous.

The environment around most of the stalls (67.8%) was clean, but only 26.7% of vendors had sufficient hand washing equipment, although 75% of the vendors were aware of the surrounding environment, principles, garbage, waste water, animals, toilets, and drains. There were no flies and vendors could see if sanitation was poor, and that without adequate washing facilities around the vendors, consumers could acquire the spread of diseases by hands.

Before each operation, the food preparer must wash their hands with clean water; however, more than half of the operators washed after going to the toilet. There were more than 60% vendors wearing clean clothing and masks and aprons.

According to the above discussion, we can see that the food safety supervision departments can clearly obtain the degree of importance attached by people to food safety and the risk links from this survey. This survey not only improves the food safety knowledge of consumers and food vendors, but also provides decision-making basis for the food safety supervision departments to develop relevant policies and organize food safety training.

## Conclusions

The main purpose of this study was to assess food safety knowledge and attitudes of street food consumers and vendors, and food handling behavior of food vendors in Handan, China. To our knowledge, this is the first study to evaluate and report these important aspects of street food safety in China.

China’s food hygiene law was introduced in 1995, and the food safety law in 2009, with stricter food safety terms and further conditions adopted in 2015. Most of the aforementioned concerns, and others discussed in this paper, are addressed in these regulations. However, the current study shows this has not yet transformed food handler knowledge, attitudes, and behavior. This could be a consequence of their relatively poor education levels, which could exclude them from certain communication channels used by the CFDA (China Food Drug Administration).

Specific recommendations from this study are as follows:

- Street food vendor training should be prioritized to improve the safety of street food.
- Other policies and measures should also be promulgated to improve the food safety knowledge, attitudes, and behavior of vendors in Handan.
- Steps should be taken to improve street food stall operating conditions and facilities, e.g., providing clean protected structures, access to potable water, and efficient waste collection and disposal systems.

Handan, the city surveyed in this paper, is a typical third-tier city with a Gross Domestic Product (GDP) of about RMB 300 billion and a population of over 1 million, which is one of the 70 cities with similar scale in China. This research can help CFDA in similar cities become more scientific in the formulation of policies with regard to food hygiene.

## Additional files

Additional file 1: 100 street vendors. Data-100 street vendors. (XLS 59 kb)

Additional file 2: 240 consumers. Data-240 consumers. (XLS 149 kb)

Additional file 3: 90 street vending stalls. Data-90 street vending stalls. (XLS 43 kb)

## Data Availability

The datasets used and/or analysed during the current study are available from the corresponding author on reasonable request.

## Abbreviations

CFDA: China Food and Drug Administration
CT: Congtai
FAO: Food and Agriculture Organization
FX: Fuxing
GDP: Gross Domestic Product
HIDZ: High-tech Industries Development Zone
HS: Hanshan
